# Severe Hereditary Hypofibrinogenemia in Pregnancy: A Case Report of a Novel Obstetrical Management with Thromboelastometry Guided Fibrinogen Supplementation

**DOI:** 10.3390/diagnostics15131671

**Published:** 2025-06-30

**Authors:** Grigorios Karampas, Konstantinos Karkalemis, Anastasia Bagiasta, Maria-Ekaterini Lefaki, Dimitra Metallinou, Chryssoula Staikou, Zoi Iliodromiti, Rozeta Sokou, Kassandra Tataropoulou, Theodora Boutsikou, Makarios Eleftheriades, Nikolaos Vlahos, Panagiotis Christopoulos, Marianna Politou

**Affiliations:** 1 Second Department of Obstetrics and Gynaecology, “Aretaieio” University Hospital, Medical School, National and Kapodistrian University of Athens, PC 11528 Athens, Greece; kark.karkalemis@gmail.com (K.K.); abagiasta@hotmail.com (A.B.); makarios@hotmail.co.uk (M.E.); nfvlahos@gmail.com (N.V.); dr_christopoulos@yahoo.gr (P.C.); 2 Laboratory of Heamatology, Blood Transfusion Unit, “Aretaieio” University Hospital, Medical School, National and Kapodistrian University of Athens, PC 11528 Athens, Greece; mklefaki@gmail.com (M.-E.L.); mpolitou10@gmail.com (M.P.); 3 Department of Midwifery, School of Health and Care Sciences, University of West Attica, PC 12243 Athens, Greece; dmetallinou@uniwa.gr; 4 First Department of Anesthesiology, “Aretaieio” University Hospital, Medical School, National and Kapodistrian University of Athens, PC 11528 Athens, Greece; c_staikou@yahoo.gr; 5 Neonatal Department, “Aretaieio” University Hospital, Medical School, National and Kapodistrian University of Athens, PC 11528 Athens, Greece; ziliodromiti@yahoo.gr (Z.I.); sokourozeta@yahoo.gr (R.S.); theobtsk@gmail.com (T.B.); 6 Neonatal Intensive Care Unit, Second Department of Pediatrics, “Aglaia Kyriakou” Children’s Hospital, Medical School, National and Kapodistrian University of Athens, PC 11573 Athens, Greece

**Keywords:** hereditary hypofibrinogenemia, pregnancy, ROTEM^®^, FIBTEM^®^, NATEM^®^

## Abstract

**Background and Clinical Significance**: Hereditary Fibrinogen Disorders (HFDs) are a group of rare, inherited coagulation disorders with a wide spectrum of clinical presentations, ranging from asymptomatic cases to severe bleeding or thrombotic events. Among these, hereditary hypofibrinogenemia (HH) poses particular challenges in obstetric care due to its unpredictable course and limited evidence-based guidelines. **Case Presentation**: This case report describes the novel obstetrical management of a 37 years old multiparous woman with severe HH (SHH) guided not only by fibrinogen levels but also by rotational thromboelastometry (ROTEM^®^), a global test of hemostasis using specific parameters such as FIBTEM^®^ and NATEM^®^ assays. Despite persistent low fibrinogen levels during labor and peripartum (<100 mg/dL), favorable maternal and neonatal outcomes were achieved by relying on ROTEM^®^-based parameters to guide clinical decisions. **Conclusions**: Current recommendations for managing pregnancies in women with HFDs are largely based on expert consensus and exclusively use fibrinogen levels. This case supports the use of specific assays (FIBTEM^®^ and NATEM^®^) of the ROTEM^®^ global test of hemostasis as valuable tools in the obstetric management of women with HH. The use of FIBTEM^®^ and NATEM^®^ assays could provide individualized perinatal care, avoiding unnecessary therapeutic interventions and aiming for optimal perinatal outcomes.

## 1. Introduction

Fibrinogen is a glycoprotein that plays a crucial role in hemostasis and clot formation. It is activated by Thrombin or Factor II (FIIa), producing fibrin monomers that are pivotal in clot formation. It also facilitates the aggregation of activated platelets during the coagulation process [1]. However, in the early stages of pregnancy, fibrinogen plays an important role in the development of fetal-maternal vascularization by supporting cytotrophoblast proliferation and expansion, resulting in normal placental formation and function [2,3].

Hereditary Fibrinogen Disorders (HFDs) are rare inherited conditions classified as quantitative, qualitative, or mixed based on fibrinogen activity and levels [4]. Quantitative types include afibrinogenemia (no fibrinogen) and hypofibrinogenemia (low fibrinogen), which are graded as mild (>100 mg/dL), moderate (50–90 mg/dL), or severe (<50 mg/dL). Qualitative disorders involve dysfunctional fibrinogen despite normal levels (dysfibrinogenemia) or both low levels and dysfunction (hypodysfibrinogenemia) [5,6].

The prevalence of HFDs is unclear due to their rarity and often mild or absent symptoms, especially in hypofibrinogenemia. Symptoms range from mild bleeding to severe hemorrhage or thrombosis, depending on the type. Women with HFDs face increased risks during pregnancy, including miscarriage, placental abruption, and severe postpartum bleeding [2,7]. The management of pregnant women with HFDs is still based on expert opinions and exclusively on fibrinogen levels [6].

In this study, we report the obstetrical management of a 37 year old multiparous woman with severe hereditary hypofibrinogenemia (SHH) monitored and treated in our institution, introducing a novel management of SHH during pregnancy directed not only by the fibrinogen level but mainly by a more holistic assessment of the coagulation profile, using specific parameters of the rotational thromboelastometry (ROTEM^®^) global test of hemostasis, such as the fibrin-based extrinsically activated test with tissue factor and the platelet inhibitor cytochalasin D (FIBTEM^®^) and the non-activated thromboelastometry (NATEM^®^) assays [8].

## 2. Case Presentation

A 37-year-old gravida 4, para 1 woman with a BMI of 20.3 kg/m^2^ presented to our institution for an outpatient appointment during her fourth pregnancy (Table 1). According to the patient’s medical history, she was diagnosed with SHH during an investigation of two first-trimester miscarriages and a subsequent singleton pregnancy. Her medical history was unremarkable, without significant menstrual bleeding or episodes of severe bleeding during childhood or adulthood.

The patient’s obstetric history included two first-trimester miscarriages and a consequent singleton full-term pregnancy delivered by cesarean section (CS). During the third pregnancy and delivery, which were monitored and carried out at a different institution, no complications were reported. The woman’s fibrinogen levels were closely monitored, and she received 2 g fibrinogen supplementation intravenously (IV) every two weeks throughout the pregnancy and an additional dose of 6 g fibrinogen concentrate (FC) IV the day prior to the elective CS.

During the fourth pregnancy, which was followed up at our institution, a different approach was applied. Fibrinogen levels were closely monitored every two to four weeks, along with FIBTEM and NATEM assays (Table 2 and Table 3). The NATEM assay provides a holistic assessment of coagulation potential, including platelets, coagulation factors, and fibrinogen, without the use of tissue factorwhile the FIBTEM assay measures fibrin-based clot formation by inhibiting platelet contribution using cytochalasin D, isolating fibrinogen function [8]. Although fibrinogen levels fluctuated from 28 mg/dL before pregnancy to 69.1 mg/dL in the first trimester (Table 1), reaching its highest level at the beginning of the third trimester (100.5 mg/dL), the FIBTEM and NATEM assays, especially the maximum clot firmness (MCF), remained within the normal range during pregnancy; thus, no fibrinogen supplementation was administered (Table 1 and Table 2). The woman had an uncomplicated pregnancy without any incidence of hemorrhage, fetal growth restriction (FGR) or abnormal Doppler parameters.

At 37 + 1 weeks of gestation, the fibrinogen level in plasma was 68.8 mg/dL. Replacement therapy with 1.5 g FC was initiated, setting the optimal peripartum fibrinogen level at 100 mg/dL. The administered dose of FC was calculated according to the following formula: dose (mg/kg) = [target level (mg/dL)-measured level (mg/dL)/0.017 g/L]. At 37 + 6 weeks of gestation, the patient was readmitted to our hospital for a new coagulation profile workup. On admission, the FIBTEM and NATEM assays were normal, and the fibrinogen level was 79.5 mg/dL. An additional dose of 1.5 g of FC was administered, and the fibrinogen level in plasma was counted once again in the afternoon at 95.4 mg/dL. Due to the high risk for obstetrical hemorrhage during the second CS, the fibrinogen level in plasma was re-counted the next morning and was 88.0 mg/dL. Although the woman received two additional doses of 1.5 g FC, the fibrinogen level remained low at 82.3 mg/dL. As bolus administration of high-dose FC could expose the woman to increased thrombotic risk, it was decided that an additional dose of 2 g FC would be administered one hour prior to the elective CS, the next morning, setting the optimal fibrinogen level in serum at 100 mg/dL. A backup dose of 1 g FC was additionally administered to the woman during CS in the case of suspected disseminated bleeding. The fibrinogen level was calculated once more just before the cesarean section at 97.2 mg/dL.

In the operating room, apart from standard monitoring (electrocardiography, heart rate, non-invasive blood pressure, oximetry), a radial arterial line was placed for intraoperative continuous blood pressure monitoring and blood sampling. Preparation for CS also included the administration of the antifibrinolytic tranexamic acid 1 g and, if needed, a 30 min infusion of 1 g FC according to guidelines and the management plan of the multidisciplinary team (hematologist, obstetrician, anesthesiologist). Cefoxitine (2 g), metoclopramide (10 mg), and cimetidine (200 mg) were also administered intravenously. Rapid sequence induction of general anesthesia and tracheal intubation were performed with thiopental (500 mg and succinylcholine 100 mg). Anesthesia was maintained with sevoflurane 1% in an O_2_/N_2_O mixture (FiO_2_:0.5), and rocuronium was used for neuromuscular blockade. After delivery, uterotonics (oxytocin 20 IU and ergometrine 0.2 mg) were administered, and fentanyl (total dose: 250 mcg) was used for analgesia. One unit of fresh frozen plasma (FFP) and 1 g of FC were administered to diminish mild blood oozing. A blood sample was sent for assessment of fibrinogen levels, which remained relatively stable (95.7 mg/dL). The parturient received 2 L of crystalloids and remained hemodynamically stable throughout the procedure. As recommended, large crystalloid volumes were avoided to reduce the risk of dilutional coagulopathy [9]. The CS was successfully completed after 40 min, with an estimated blood loss of 400 mL. Recovery from anesthesia was uneventful, and sufficient postoperative analgesia was provided with paracetamol 1 g and morphine 6 mg IV. No hemorrhagic complications occurred during surgery or throughout the postpartum period.

Postoperatively, no additional supplementation therapy was administered to the mother, and 12 h after the CS, a prophylactic dose of low-molecular-weight heparin (LMWH) was initiated once every day. Fibrinogen levels as well as FIBTEM and NATEM assays were measured systematically postpartum, with fibrinogen levels of 105.3 mg/dL, 128.9 mg/dL, 106.4 mg/dL, and 94.2 mg/dL on the first, second, fourth, and seventh postpartum days, respectively (Table 2). During the entire postpartum period, FIBTEM and NATEM assays remained within the normal range (Table 2 and Table 3), guiding the decision for the use or non-use of additional therapy. The woman was eventually discharged 2 days after delivery, presenting an uncomplicated postpartum course, and was followed up during the first month with regular outpatient visits on days 10 and 30 after discharge.

A healthy full-term male neonate weighing 3370 g (APGAR scores 9^1^-10^5^-10^10^) was born and admitted to a Level-II Neonatal Intensive Care Unit (NICU) and transferred to a Level III NICU after birth. Further evaluation of the neonate in the NICU, focusing on the coagulation status, revealed that the neonate inherited his mother’s hypofibrinogenemia, with a fibrinogen level of 35 mg/dL on the first day of life, which remained lower than 50 mg/dL during hospitalization. The highest fibrinogen level observed was 40 mg/dL, and no supplementation therapy was administered. The neonate had an uncomplicated stay in the NICU and was discharged on the fourth day of life.

## 3. Discussion

Hereditary hypofibrinogenemia (HH) is a rare, autosomal dominant disorder that affects both sexes equally [10]. The disorder is caused by mutations in three different genes located on chromosome 4 that form three paired polypeptide chains ( alpha, beta, and gamma) that produce the hexamer of fibrinogen glycoprotein [11,12]. Mutations in any of the three fibrinogen genes can result in distinct fibrinogen-related disorders, each characterized by unique clinical manifestations [13]. Our insight into the genetic basis of hypofibrinogenemia was further extended by a recent study by Mohsenian et al. [14], who utilized data from the Prospective Rare Bleeding Disorders Database (PRO-RBDD). Thus, most patients with hypofibrinogenemia carry autosomal dominant missense or null mutations, mostly in the fibrinogen gamma gene [14,15].

**Figure 1:**
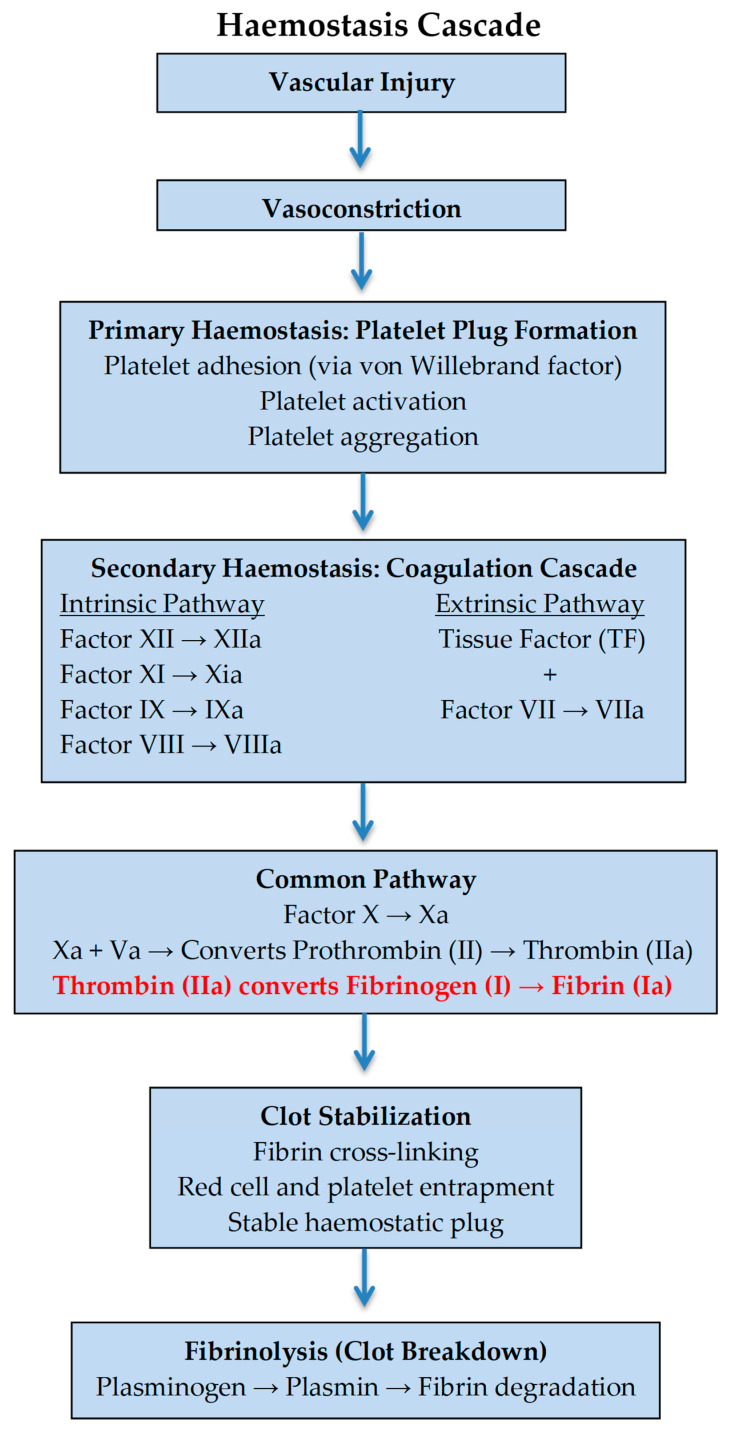
Hemostasis cascade highlighting (red) impaired clot formation due to low fibrinogen levels in cases of hereditary hypofibrinogenemia.

Although many women with HH remain asymptomatic and are diagnosed incidentally during routine coagulation testing during pregnancy, clinical manifestations can vary widely. Some may experience a higher tendency for cutaneous bleeding or other mild bleeding symptoms, such as epistaxis or menorrhagia, while others are at risk for severe bleeding or thrombotic events based on fibrinogen deficiency (Figure 1) [16,17]. Although SHH was already known in our case, many cases of HFDs can be diagnosed for the first time during pregnancy, making the differential diagnosis of the specific subtype of fibrinogen disorder a challenge. In such cases, the utility of the FIBTEM assay is crucial, as an abnormal parameter of the FIBTEM assay is unlikely to be present in HH compared to dysfibrinogenemia diagnosis, in which the parameters of the FIBTEM assay are ambiguous [18].

Although HH does not typically interfere with conception or early implantation, fibrinogen plays a key role in placental development and the establishment of a stable fetal-maternal vascular connection [2,19]. Adverse obstetric outcomes may include early miscarriages (typically between 5 and 8 weeks of gestation), FGR, preeclampsia, placental abruption, preterm delivery, and life-threatening postpartum hemorrhage (PPH) [5,11,20,21]. Consequently, women with HH, especially those with SHH, are at an increased risk of severe complications during pregnancy, from conception to labor and postpartum [20]. Therefore, similar cases should always be managed in a tertiary center, as they require a multidisciplinary team approach, including experienced obstetricians, hematologists, anesthesiologists, and neonatologists, close surveillance, and possible supplementation therapies.

The existing literature emphasizes that the key to managing high-risk pregnancies in women with HFDs is the regular monitoring of fibrinogen levels and appropriate supplementation therapy [5,6]. Fibrinogen supplementation therapy includes three primary options: fresh frozen plasma (FFP), cryoprecipitate, and fibrinogen concentrate (FC), with FC being the most appropriate choice [22,23,24,25]. Current perspectives on management (Delphi consensus) recommend monthly fibrinogen checks during pregnancy, with levels maintained between 50 and 100 mg/dL to prevent miscarriage or other complications, while during the peripartum period and labor, levels should be kept above 150 mg/dL. The advantages of FC over FFP or cryoprecipitate are that FC does not require ABO compatibility, is less likely to cause allergic reactions or lead to pathogen transfusion, and spares the infusion of other additional coagulant factors, such as factor VIII and von Willebrand factor. Most importantly, in cases of postpartum hemorrhage, when the administration of fluids is extensive, FC offers the advantage that it can be administered in a more concentrated volume, as each unit consists of 50 mL of fibrinogen solution at a concentration of 20 mg/mL, corresponding to a total administration of 1 g/unit, which leads to a serum fibrinogen elevation of approximately 30 mg/dL. Finally, if thromboprophylaxis is needed postpartum, LMWH is advised based on the woman’s bleeding/thrombotic individual risk [6,25].

The mode of delivery and labor management in cases of HH during pregnancy is of high importance, since due to the autosomal dominant nature of the disease, the embryo or neonate is considered to be at high risk for any form of bleeding or hemorrhagic complication, including birth injuries such as cephalohematoma, subdural or subarachnoid hemorrhage, and the most serious intraventricular hemorrhage (IVH) [26]. Therefore, even though the existing literature does not exclude vaginal delivery in pregnant women with HH, it is advised that it should be performed or supervised by a senior obstetrician, as the possibility of assisted vaginal delivery, by the use of vacuum or forceps, would increase the risk of hemorrhagic complications for both the mother and the neonate [27]. For the same reason, novel fetal monitoring methods during labour, such as fetal scalp blood sampling (FBS) or even internal cardiotocography, which could potentially lead to fetal injury or bleeding, are strongly discouraged, even when the CTG tracing is suspicious or abnormal [28]. Consequently, a holistic evaluation of the woman’s obstetric history, physical characteristics such as height, weight and BMI, along with current pregnancy details—including ultrasound measures like estimated fetal weight (EFW), amniotic fluid levels (AFI), and Doppler findings—as well as any medical conditions like diabetes, is important for optimal pregnancy and labour management.

In our case, two totally different therapeutic strategies were applied to the same woman in her two pregnancies with similar, uncomplicated outcomes. Specifically, based only on fibrinogen levels, the woman received during her first full-term pregnancy 2 g of FC every two weeks as supplementation therapy, targeting a fibrinogen level ≥ 100 mg/dL and an additional dose of 6 g FC peripartum. On the contrary, based on the FIBTEM assay, we applied a different approach as the woman received no supplementation therapy during her second full-term pregnancy except for the peripartum administration of 6.5 g of FC divided into 4 doses within 24 h and one unit of FFP intraoperatively in combination with 1 g FC. Though in both pregnancies the perinatal outcome was excellent, it is worth mentioning that during the second pregnancy the woman avoided the IV treatment with FC twice every month, resulting in less need for hospitalization and the cost of treatment.

Moreover, as it is well documented that the FIBTEM assay is useful in the perioperative management of fibrinogen replacement therapy in patients with HDFs, fibrinogen administration peripartum in our case was mainly guided by the FIBTEM assay [29,30]. For the FIBTEM assay, clot amplitude 10 min after clotting time (A10) and MCF were recorded and taken into special consideration for decision making (Table 2). The MCF parameter reflects the total firmness of the clot formed in the presence of cytochalasin D, which inhibits the platelets and provides wider information than fibrinogen concentration, as it can measure the elasticity of fibrin-based clot, which depends not only on fibrinogen but on other coagulation factors such as Factor XIII [31]. As mentioned before, there is a broad consensus that maintaining fibrinogen levels ≥ 150 mg/dL during labour minimizes complications [6]. However, in our case, levels remained below 100 mg/dL despite administering 6.5 g of FC within 24 h antepartum. To our knowledge, this is the first reported case where a high FC dose had minimal effect on serum levels. Possible explanations include increased serum/plasma volume and glomerular filtration rates during pregnancy. In such cases, higher FC doses should be used cautiously due to the potential thrombotic risk without added hemostatic benefit. Consequently, based on a normal FIBTEM assay, no additional dose of FC was administered to the woman antepartum without any major hemorrhagic event during the CS.

As this is the first time that FIBTEM, and not fibrinogen levels, are used for decision making in a case of SHH during pregnancy, its effectiveness in predicting the clinical phenotypes of these disorders and an uncomplicated perinatal outcome must be confirmed by larger prospective studies [8,18,30,32]. Pregnancy is a hypercoagulable state, as the body naturally enhances its ability to form clots in preparation for delivery [33]. This physiological change affects various hemostatic parameters, including those measured by the ROTEM^®^ test [33]. In general, there is an upregulation of all FIBTEM and NATEM parameters, with, for example, increased FIBTEM-MCF, shorter clot formation time (CFT), and higher clotting time (CT) [33,34]. These alterations are more pronounced in the third trimester, and the reference ranges should be adjusted for pregnant women to avoid misinterpretation or underestimating the bleeding risk [33]. Nevertheless, there is no universal consensus regarding the reference ranges of FIBTEM and NATEM parameters during pregnancy; not all parameters are adequately studied in pregnancy, and in many studies, absolute ranges often overlap with those in non-pregnant [34,35,36]. For these reasons, it is strongly recommended that institution-specific validation is encouraged, especially because ROTEM^®^ reference values can vary by device. Consequently, as in our case, a holistic evaluation of each woman’s coagulation status is recommended, including systematic follow-up of fibrinogen levels and assays of the ROTEM^®^ global test of hemostasis.

Thus, parameters of the ROTEM^®^ test might provide not only a diagnostic tool in unknown cases of HFDs during pregnancy but also a decision-making parameter for individualized perinatal care, avoiding unnecessary treatment strategies during pregnancy or increased thrombotic risk peripartum.

## 4. Conclusions

In conclusion, HH is a rare obstetrical challenge with many undiscovered and unpredictable aspects. Consensus among experts supports maintaining fibrinogen concentration at or above 100 mg/dL during pregnancy, 150 mg/dL during labour, and implementing postpartum thromboprophylaxis with low-molecular-weight heparin (LMWH) when clinically indicated based on patients’ bleeding/thrombotic individual risk, to minimize the risk for perinatal complications. The favorable outcomes observed in both pregnancies of the presented case with SHH underscore the importance of re-evaluating current management strategies for such rare obstetric cases. Our case raises important questions about whether management of cases with HH during pregnancy should be based solely on fibrinogen levels as the only laboratory parameter guiding the supplementation therapy, or a more individualized risk assessment and therapeutic strategy should be implemented. Thus, a more holistic approach to women’s coagulation status is proposed, including not only fibrinogen levels but also other parameters such as FIBTEM and NATEM assays. The use of the FIBTEM assay, which mainly guided the need for fibrinogen administration in our case, provides a novel approach for monitoring and treatment of women with HH during pregnancy, and needs to be further confirmed by larger prospective studies.

## Figures and Tables

**Table 1:** Maternal-neonatal-pregnancy characteristics and perinatal outcomes.

Pregnancy	Age (Years)	Gravidity	Parity	Medical History	Labour Week	Mode of Delivery	Anaesthesia or Analgesia Method	Birth Weight (g)	Apgar Score	Complications
1st	33	1	0	-	-	-	-	-	-	Misscarriage at 6th week
2nd	34	2	0	-	-	-	-	-	-	Misscarriage at 8th week
3st	35	3	1	-	40 + 1 w	ECS	GA	3350	9^1^ 10^5^	-
4nd	37	4	1	-	38 + 1 w	ECS	GA	3370	9^1^ 10^5^	-

ECS: elective caesarean section; GA: general anesthesia.

**Table 2:** Weeks of labor, fibrinogen levels, and FIBTEM assay throughout pregnancy, antepartum (AP), and postpartum (PP).

Week	Fibrinogen	FIBTEM						
	(mg/dL) 180–450	CT (sec) 38–62	CFT (sec) 30–70	A10 (mm) 7–23	A20 (mm) 8–24	MCF (mm) 9–25	LI30 (%) <15%	LI60 (%) <15%
8 + 3	69.1	89	-	11	13	14	100	100
16 + 3	78.2	102	-	9	11	13	100	100
21 + 4	69.0	98	-	8	9	10	100	100
23 + 0	67.6	91	-	8	10	10	100	100
24 + 0	93.2	91	-	13	14	14	100	100
26 + 1	63.2	94	-	8	10	13	100	100
29 + 0	77.4	90	-	10	11	13	100	100
31 + 3	100.5	90	2516	15	18	21	100	100
34 + 1	70.8	77	-	11	12	15	100	98
36 + 0	68.5	78	-	9	11	14	100	100
37 + 1	68.8	128	-	9	12	14	100	100
37 + 6 10:25	79.5	83	-	9	11	14	100	100
37 + 6 17:03	95.4	-	-	-	-	-	-	-
38 + 0	88.0	-	-	-	-	-	-	-
38 + 1	82.3	-	-	-	-	-	-	-
38 + 2 AP	97.2	-	-	-	-	-	-	-
38 + 2 10:53 PP	94.0	-	-	-	-	-	-	-
38 + 2 13:38 PP	103.6	89	-	11	13	15	100	100
38 + 2 18:54 PP	95.7	-	-	-	-	-	-	-
1st day PP	105.3	59	3998	14	16	21	100	100
2nd day PP	128.9	-	-	-	-	-	-	-
4th day PP	106.4	72	3533	15	17	20	100	100
7th day PP	94.2	87	-	12	13	15	100	100

FIBTEM, fibrin-based extrinsically activated test with tissue factor and the platelet inhibitor cytochalasin D; CT, clotting time; CFT, clot formation time; A10, clot amplitude 10 min after CT; A20, clot amplitude 20 min after CT; MCF, maximum clot firmness; LI30 (%): lysis index at 30 min; LI60 (%): lysis index at 60 min.

**Table 3:** Weeks of labor, fibrinogen levels, and NATEM assay throughout pregnancy, antepartum (AP), and postpartum (PP).

Week	Fibrinogen	NATEM							
	(mg/dL) 180–450	CT (sec) 300–999	CFT (sec) 150–700	Alp (α-angle) 30–70	A10 (mm) 35–60	A20 (mm) 35–60	MCF (mm) 40–65	LI30 (%) <15%	LI60 (%) <15%
8 + 3	69.1	717	198	54	40	50	54	100	99
16 + 3	78.2	555	167	59	42	51	54	100	99
21 + 4	69.0	293	169	59	42	51	53	100	97
23 + 0	67.6	467	186	56	40	49	52	100	97
24 + 0	93.2	588	144	62	47	55	57	100	97
26 + 1	63.2	529	148	61	45	53	55	100	
29 + 0	77.4	535	151	61	46	54	56	100	97
31 + 3	100.5	582	129	65	53	60	62	100	98
34 + 1	70.8	487	152	61	45	53	55	100	98
36 + 0	68.5	474	177	57	42	51	54	100	99
37 + 1	68.8	444	150	62	43	52	54	100	100
37 + 6 10:25	79.5	-	-	-	-	-	-	-	-
37 + 6 17:03	95.4	-	-	-	-	-	-	-	-
38 + 0	88.0	-	-	-	-	-	-	-	-
38 + 1	82.3	-	-	-	-	-	-	-	-
38 + 2 AP	97.2	-	-	-	-	-	-	-	-
38 + 2 10:53 PP	94.0	-	-	-	-	-	-	-	-
38 + 2 13:38 PP	103.6	529	161	59	45	53	57	100	100
38 + 2 18:54 PP	95.7	-	-	-	-	-	-	-	-
1st day PP	105.3	243	286	49	44	56	61	100	100
2nd day PP	128.9	-	-	-	-	-	-	-	-
4th day PP	106.4	605	194	57	50	60	63	100	98
7th day PP	94.2	288	124	66	52	59	60	100	96

NATEM, non-activated thromboelastometry; CT, clotting time; CFT, clot formation time; Alp, α-angle; A10, clot amplitude 10 min after CT; A20, clot amplitude 20 min after CT; MCF, maximum clot firmness; LI30 (%): lysis index at 30 min; LI60 (%): lysis index at 60 min.

## Data Availability

Data is contained within the article.

## Abbreviations

The following abbreviations are used in this manuscript:

HFDs	Hereditary Fibrinogen Disorders
HH	Hereditary hypofibrinogenemia
SHH	Severe Hereditary hypofibrinogenemia
ROTEM^®^	Rotational thromboelastometry
FIBTEM^®^	Fibrin-based thromboelastometry
NATEM^®^	Non-activated thromboelastometry
CS	Cesarian Section
IV	Intravenously
FC	Fibrinogen Concentrate
MCF	Maximum clot firmness
FGR	Fetal growth restriction
FFP	Fresh frozen plasma
LMWH	Low-molecular-weight heparin
NICU	Neonatal Intensive Care Unit
PPH	Post-Partum Hemorrhage

## References

[1] Inbal A., Muszbek L. (2003). Coagulation Factor Deficiencies and Pregnancy Loss. Semin. Thromb. Hemost..

[2] Zhang Y., Zuo X., Teng Y. (2020). Women With Congenital Hypofibrinogenemia/Afibrinogenemia: From Birth to Death. Clin. Appl. Thromb..

[3] Mosesson M.W., Siebenlist K.R., Meh D.A. (2001). The Structure and Biological Features of Fibrinogen and Fibrin. Ann. N. Y. Acad. Sci..

[4] Acharya S.S., Dimichele D.M. (2008). Rare Inherited Disorders of Fibrinogen. Haemophilia.

[5] Casini A., De Moerloose P., Neerman-Arbez M. (2016). Clinical Features and Management of Congenital Fibrinogen Deficiencies. Semin. Thromb. Hemost..

[6] Casini A., de Moerloose P. (2016). Management of Congenital Quantitative Fibrinogen Disorders: A Delphi Consensus. Haemophilia.

[7] Valiton V., Hugon-Rodin J., Fontana P., Neerman-Arbez M., Casini A. (2019). Obstetrical and Postpartum Complications in Women with Hereditary Fibrinogen Disorders: A Systematic Literature Review. Haemophilia.

[8] Drotarova M., Zolkova J., Belakova K.M., Brunclikova M., Skornova I., Stasko J., Simurda T. (2023). Basic Principles of Rotational Thromboelastometry (ROTEM(^®^)) and the Role of ROTEM-Guided Fibrinogen Replacement Therapy in the Management of Coagulopathies. Diagnostics.

[9] Kietaibl S., Ahmed A., Afshari A., Albaladejo P., Aldecoa C., Barauskas G., De Robertis E., Faraoni D., Filipescu D.C., Fries D. (2023). Management of Severe Peri-Operative Bleeding: Guidelines from the European Society of Anaesthesiology and Intensive Care: Second Update 2022. Eur. J. Anaesthesiol. EJA.

[10] Agarwal M.B., Sanzgiri P.S., Bhanotra P.C., Rao S., Mehta B.C., Shah M.D. (1981). Congenital Disorders of Fibrinogen. J. Postgrad. Med..

[11] Frenkel E., Duksin C., Herman A., Sherman D.J. (2004). Congenital Hypofibrinogenemia in Pregnancy: Report of Two Cases and Review of the Literature. Obstet. Gynecol. Surv..

[12] Tiscia G.L., Margaglione M. (2018). Human Fibrinogen: Molecular and Genetic Aspects of Congenital Disorders. Int. J. Mol. Sci..

[13] Wypasek E., Klukowska A., Zdziarska J., Zawilska K., Treliński J., Iwaniec T., Mital A., Pietrys D., Sydor W., Neerman-Arbez M. (2019). Genetic and Clinical Characterization of Congenital Fibrinogen Disorders in Polish Patients: Identification of Three Novel Fibrinogen Gamma Chain Mutations. Thromb. Res..

[14] Mohsenian S., Palla R., Menegatti M., Cairo A., Lecchi A., Casini A., Neerman-Arbez M., Asselta R., Scardo S., Siboni S.M. (2024). Congenital Fibrinogen Disorders: A Retrospective Clinical and Genetic Analysis of the Prospective Rare Bleeding Disorders Database. Blood Adv..

[15] Neerman-Arbez M., Casini A. (2018). Clinical Consequences and Molecular Bases of Low Fibrinogen Levels. Int. J. Mol. Sci..

[16] Soares A.W., Maia M., Santo J.E., Costa A.P., Pereira A., Catarino C. (2020). Hypofibrinogenaemia: A Case of Spontaneous Bleeding and Central Venous Thrombosis in the Same Lifetime. Eur. J. Case Rep. Intern. Med..

[17] Mumford A.D., Ackroyd S., Alikhan R., Bowles L., Chowdary P., Grainger J., Mainwaring J., Mathias M., O’Connell N. (2014). Guideline for the Diagnosis and Management of the Rare Coagulation Disorders. Br. J. Haematol..

[18] Georgiadou P., Sokou R., Tsantes A.G., Parastatidou S., Konstantinidi A., Houhoula D., Kokoris S., Iacovidou N., Tsantes A.E. (2022). The Non-Activated Thromboelastometry (NATEM) Assay’s Application among Adults and Neonatal/Pediatric Population: A Systematic Review. Diagnostics.

[19] Goodwin T.M. (1989). Congenital Hypofibrinogenemia in Pregnancy. Obstet. Gynecol. Surv..

[20] Iwaki T., Castellino F.J. (2005). Maternal Fibrinogen Is Necessary for Embryonic Development. Curr. Drug Targets.

[21] Ness P.M., Budzynski A.Z., Olexa S.A., Rodvien R. (1983). Congenital Hypofibrinogenemia and Recurrent Placental Abruption. Obstet. Gynecol..

[22] Cai H., Liang M., Yang J., Zhang X. (2018). Congenital Hypofibrinogenemia in Pregnancy: A Report of 11 Cases. Blood Coagul. Fibrinolysis.

[23] Bornikova L., Peyvandi F., Allen G., Bernstein J., Manco-Johnson M.J. (2011). Fibrinogen Replacement Therapy for Congenital Fibrinogen Deficiency. J. Thromb. Haemost..

[24] Bevan D.H. (2009). Cryoprecipitate: No Longer the Best Therapeutic Choice in Congenital Fibrinogen Disorders?. Thromb. Res..

[25] Watanabe K., Nakajima Y., Sakanashi M. (2013). Perioperative Management of a Patient with Congenital Hypofibrinogenemia Who Underwent Myomectomy and Cesarean Section. J. Jpn. Soc. Clin. Anesth..

[26] Diguisto C., Baker E., Stanworth S., Collins P.W., Collis R.E., Knight M. (2024). Management and Outcomes of Women with Low Fibrinogen Concentration during Pregnancy or Immediately Postpartum: A UK National Population-Based Cohort Study. Acta Obstet. Gynecol. Scand..

[27] Huq F.Y., Kadir R.A. (2011). Management of Pregnancy, Labour and Delivery in Women with Inherited Bleeding Disorders. Haemophilia.

[28] Teraoka Y., Miyoshi H., Oshima K., Urabe S., Tanaka N., Kudo Y. (2017). Prenatal and Peripartum Management of Patients with Hypofibrinogenemia Resulted in Two Successful Deliveries. Case Rep. Obstet. Gynecol..

[29] Khunakanan S., Akaraborworn O., Sangthong B., Thongkhao K. (2019). Correlation between Maximum Clot Firmness in FIBTEM and Fibrinogen Level in Critical Trauma Patients. Crit. Care Res. Pract..

[30] Zhou J., Xin Y., Ding Q., Jiang L., Chen Y., Dai J., Lu Y., Wu X., Liang Q., Wang H. (2016). Thromboelastography Predicts Risks of Obstetric Complication Occurrence in (Hypo)Dysfibrinogenemia Patients under Non-Pregnant State. Clin. Exp. Pharmacol. Physiol..

[31] Treliński J., Pachniewska K., Matczak J., Robak M., Chojnowski K. (2016). Assessment of Selected ROTEM Parameters, Kinetics of Fibrinogen Polymerization and Plasmin Amidolytic Activity in Patients with Congenital Fibrinogen Defects. Adv. Clin. Exp. Med. Off. Organ. Wroc. Med. Univ..

[32] Schöchl H., Cotton B., Inaba K., Nienaber U., Fischer H., Voelckel W., Solomon C. (2011). FIBTEM Provides Early Prediction of Massive Transfusion in Trauma. Crit. Care.

[33] Bagot C.N., Leishman E., Onyiaodike C.C., Jordan F., Freeman D.J. (2017). Normal pregnancy is associated with an increase in thrombin generation from the very early stages of the first trimester. Thromb. Res..

[34] Ronenson A.M., Shifman E.M., Kulikov A.V., Raspopin Y.S., Görlinger K., Ioscovich A.M., Tikhova G.P. (2022). Rotational Thromboelastometry Reference Range during Pregnancy, Labor and Postpartum Period: A Systematic Review with Meta-Analysis. J. Obstet. Anaesth. Crit. Care.

[35] Lee J., Wyssusek K.H., Kimble R.M.N., Way M., van Zundert A.A., Cohen J., Rowell J., Eley V.A. (2020). Baseline parameters for rotational thromboelastometry (ROTEM^®^) in healthy pregnant Australian women: A comparison of labouring and non-labouring women at term. Int. J. Obs. Anesth..

[36] Amgalan A., Allen T., Othman M., Ahmadzia H.K. (2020). Systematic review of viscoelastic testing (TEG/ROTEM) in obstetrics and recommendations from the women’s SSC of the ISTH. J. Thromb. Haemost..

